# Electrical Characterization of Cost-Effective Screen-Printed Sensors Based on Thermoplastic Polyurethane, Polyimide, and Polyethylene Terephthalate

**DOI:** 10.3390/mi16030319

**Published:** 2025-03-10

**Authors:** Muhammad Faiz ul Hassan, Yan Wang, Kai Yang, Yading Wen, Shichao Jin, Yi Zhang, Xiaosheng Zhang

**Affiliations:** School of Integrated Circuit Science and Engineering, University of Electronic Science and Technology of China, Chengdu 611731, China; faizulhassan858@yahoo.com (M.F.u.H.); jinsco@163.com (S.J.); zhangxs@uestc.edu.cn (X.Z.)

**Keywords:** wearable electronics, flexible electronics, screen printing, strain sensors, thermoplastic polyurethane (TPU)

## Abstract

In recent years, the improvement in living standards and the corresponding increase in quality-of-life expectations have significantly increased the demand for advanced electronic products. This trend has generated great interest in human health monitoring and extensive research efforts. Flexible sensors in particular are being given preference because of their high extensibility, excellent biocompatibility properties, low weight, and low cost. In the present work, we took this idea further and designed flexible sensors using different substrates such as thermoplastic polyurethane (TPU), polyimide (PI), and polyethylene terephthalate (PET), fabricating them with silver paste ink using screen-printing technology. A uniform and homogeneous conductive layer was formed, which was identified through Scanning Electron Microscopy (SEM) analysis. Additionally, the width of the printed silver paste ink was approximately 100 µm. This study contributes to the design and fabrication of a new generation of flexible sensors for health monitoring. The results demonstrate that these sensors are technically possible as part of long-term wearable health-monitoring solutions for wearable health care technologies.

## 1. Introduction

Flexible stretchable, twistable, and bendable electronic devices can cover curved complex surfaces, which have become a great advancement in electronic devices [1,2]. Wearable sensors have many advantages including a wide range, different structures, and excellent biocompatibility [3,4]. They show significant potential in the areas of wireless flexible electronics, smart sensing robots, self-powered stretchable platforms, and small physiological signal monitoring systems [5,6]. In recent decades, with the improvement of people’s living standards and quality of life, the requirements for electronic products have significantly increased. Wearable sensors have been developed using a variety of low-cost materials [7,8]. The field of human health monitoring has gathered substantial attention from researchers around the world. Among the advancement in this domain, smart wearable devices equipped with various sensors have become particularly prominent. These flexible devices utilize micro-nanoelectronics to deliver critical health-monitoring capabilities [9,10,11]. Furthermore, for the purpose of monitoring human health and preventing cardiovascular disease, the detection of electrophysiological signals from tiny skin surface deformations requires great sensitivity over a broad sensing range [12,13]. The rapid development of microelectronic science and technology has driven significant advancements in the manufacturing processes of microelectronic devices [14]. These advancements have led to the continuous improvement of materials used for device fabrication. However, flexible sensors face challenges such as the poor electrical conductivity of flexible materials, which can impact the sensitivity and performance of the sensors. To address these challenges, extensive research has been conducted on flexible sensor sensing materials [15]. For example, integrating metal nanomaterials with excellent electrical conductivity and flexible carbon nanotube (CNT) materials has shown promise in enhancing the conductivity of flexible sensors.

The basis of sensor technology is fixed in the inherent properties of the materials, and significant advancements are often made through the meticulous selection of a flexible substrate and conductive material. The flexible substrate plays a crucial role, as it not only provides physical support, but it also has a substantial impact on the sensor’s performance, wearability, and durability [16,17].

Sensitivity is a crucial indicator for evaluating a sensor’s performance. It quantifies the responsiveness of the sensor’s output to variations in the physical quantity being measured. Sensitivity facilitates a clear assessment of the measurement’s accuracy and stability [18]. The primary criteria when selecting a flexible substrate are its flexibility and stretchability, allowing it to adapt to various bending and stretching conditions while maintaining structural integrity and functionality. Additionally, the substrate must exhibit chemical and thermal stability under diverse environmental conditions. This includes resistance to moisture, chemical corrosion, and temperature fluctuations [19,20]. For applications in the medical and wearable sectors, biocompatibility is essential to ensure safety and comfort during continued skin contact [21,22]. Although hydrogels are commonly used as substrate materials in many flexible sensors, their tendency to lose water leads to poor stability [23,24]. Consequently, stable polymer elastomers have gained favor among researchers seeking to enhance the performance of flexible sensors [25,26]. Continuous innovation in flexible substrate materials expands the possibilities for the design and application of flexible sensors. The core function of these sensors is to effectively detect and transmit electrical signals, a capability largely dependent on the conductive materials chosen [27]. Advancements in science and technology have extended the range of conductive materials from traditional metals and alloys to advanced conductive polymers and nanomaterials. Conductive materials must withstand environmental factors such as temperature changes, humidity, and chemical corrosion while maintaining excellent electrical conductivity. Metal-based and carbon-based nanomaterials in particular have garnered attention for their outstanding electrical conductivity, flexibility, and stability, showing great potential in strengthening composites [28,29,30]. Flexible sensors, especially when used in health monitoring, require a careful balance between mechanical durability and electrical sensitivity. TPU, PI, and PET substrates provide diverse levels of flexibility, thermal stability, and mechanical strength, rendering them appropriate options for various sensor applications [31,32].

The screen-printing technique, famous for its ease and scalability, facilitates the application of functional materials, such as conductive inks, onto these substrates, thereby enabling the development of sensors with detailed electrical characteristics tailored to specific health-monitoring requirements [33,34]. The inks used in screen printing are specifically formulated for the process, differing from standard paint stains [35]. These specialized inks are essential for achieving the desired results, especially when printing on diverse materials or incorporating functional elements like conductive circuits into the design [36]. The flexibility of screen printing extends to its ability to work on a wide range of substrates, making it a popular choice for various applications, from textiles to electronic components [37]. One of the key advantages of screen printing is its capacity for high-speed production, making it appropriate for large-scale manufacturing [38,39]. Additionally, the technique allows for a high level of detail and accuracy, particularly when creating complex, layered designs [40]. However, the success of the process depends on the careful selection of the appropriate ink and substrate combination and thorough testing to ensure the best possible results [41,42]. In recent years, there has been a strong focus on improving the mechanical and electrical properties of sensors that are made using these substrates. The goal is to enhance their performance in real-world situations. In order to accomplish this, researchers are conducting studies on the correlation between the substrate material, the conductive layers, and the operational environment of the sensors [43,44].

In this research, we performed a comprehensive study on the fabrication of flexible sensors using different substrates such as polyimide (PI), thermoplastic polyurethane (TPU), and polyethylene terephthalate (PET), combined with silver paste ink. These films have excellent mechanical properties to serve as flexible substrate. These films ensured that our flexible devices could endure bending and stretching conditions while preserving their structural integrity and functionality. As flexible and stretchable electrodes fully screen-printed on the substrates, they showed outstanding mechanical compliance during deformation, such as twisting, bending, and stretching.

## 2. Materials and Methods

### 2.1. Materials

The materials used in the fabrication of flexible sensors play an important role in defining their electrical and mechanical properties. The main materials used in this study were different substrates and silver paste ink. Flexible substrates play a crucial role in the performance of wearable electronics due to their exceptional ability to conform to several shapes and endure mechanical stress. In this research study, the following three substrates were used. Thermoplastic polyurethane (TPU) was purchased from Dongguan Xinhong Plastic Products Co., Ltd., Dongguan, China. The thickness of this substrate material was about 0.3 mm. TPU offers a unique combination of elasticity, transparency, and resistance. It is highly stretchable and flexible, making it suitable for wearable applications. Polyimide (PI) is a high-performance polymer with excellent thermal stability, chemical resistance, and mechanical strength. This is an ideal substrate for applications requiring high-temperature resistance and durability. The PI substrate was purchased from Shenzhen Jinlvye Technology Co., Ltd., Shenzhen, China. The thickness of the substrate was 25 μm. Polyethylene terephthalate (PET) has excellent chemical resistance with optical clarity and flexibility. It is commonly used in flexible electronics applications due to its transparency and mechanical properties. PET was purchased from Suzhou Dongxuan Plastic Products Co., Ltd., Suzhou, China. The thickness of the PET film used in this research study was 25 μm. The conductive silver paste ink was purchased from the Shanren (Zhenjiang) New Materials Technology Co., Ltd., Zhenjiang, China. We selected these materials due to their specific material properties, which are essential for wearable electronics. However, it is valuable to consider how they compare with other commonly used flexible substrates such as PDMS (polydimethylsiloxane) and ecoflex; a comparison can be found in Table S1.

### 2.2. Fabrication Method

This screen-printing process is commonly used in different applications including flexible electronics, sensors, and printed electronics devices. It allows for precise control over the pattern and is suitable for mass manufacturing due to its simplicity and productivity. Among the various fabrication techniques, the screen-printing method stands out for its ease of use and material versatility, making it a preferred choice in the production of mechanical strain sensors. Based on current material costs and the screen-printing process, the estimated cost per sensor depends on factors such as the substrate material (PET, TPU, or PI) and the complexity of the fabrication process and the equipment. This cost is significantly lower than alternative manufacturing methods for flexible sensors. For example, sensors fabricated using photolithography typically have higher production costs due to the more expensive and more intricate fabrication processes. Additionally, methods like roll-to-roll printing or laser cutting, while scalable for mass production, still entail higher initial setup costs and specialized equipment, making them less cost-effective for smaller-scale or prototype production. Compared to these methods, screen printing offers a more affordable and scalable solution, especially for wearable applications where cost-effectiveness is crucial for widespread adoption.

Here, we explored resistive-type strain sensors. Resistive strain sensors have extensive applications in sensor device design. Their use can be attributed to their straightforward structure, ease of signal acquisition, and high performance. Through employing these attributes, we can continue to develop and improve health-monitoring technologies that are both efficient and effective, ultimately enhancing our ability to monitor and maintain human health with precision and reliability.

**Figure 1:**
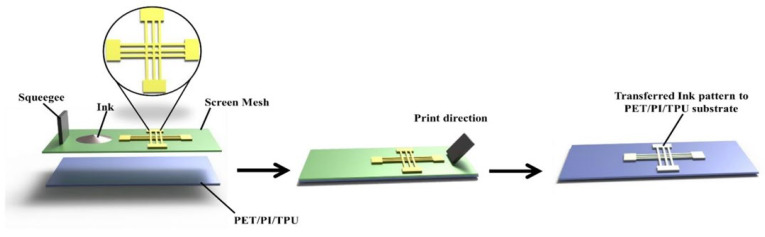
Fabrication process for transferring ink patterns to a substrate.

The process included using a screen mesh and squeegee to transfer the silver paste ink in a specific pattern, as shown in Figure 1; a more detailed description of the screen-printing process can be found in Figure S1. The fabrication process of the sensors utilized screen printing equipment supplied by Shanghai Xuanting Co., Ltd., Shanghai, China. Plasma treatment of the materials’ surface was conducted before the printing process using oxygen at a power of 50 W for 1 min. A surface pre-treatment technique, i.e., the plasma treatment, was implemented to enhance ink–substrate interactions and minimize variations due to surface energy differences; more details can be found in Figure S2. A screen mesh stencil with the desired pattern was placed over the TPU, PI, and PET substrates. The mesh contained open areas corresponding to the pattern that will be printed. The type of ink was selected based on the required properties, such as conductivity and flexibility. The ink was placed on top of the mesh in a thick layer. A squeegee with a flat flexible blade was placed at one end of the screen mesh. The squeegee was then moved across the mesh in the print direction to spread the ink. As it moved, the squeegee pressed the ink through the open areas of the mesh. The ink passed through the open areas of the screen mesh and was deposited onto the substrate, forming the desired pattern. The screen mesh was then lifted away leaving the printed ink pattern on the substrate. The transferred ink pattern on the TPU, PI, and PET substrates precisely replicated the mesh pattern.

### 2.3. Material Characterization and Measurement

Scanning Electron Microscopy (SEM) images were captured using a scanning electroscope from Phenom Scientific Instruments Shanghai Co., Ltd., Shanghai, China. SEM was also used to analyze the surface morphology and the quality of the printed patterns. High-resolution images were obtained to measure the widths of the printed lines and to inspect the integrity of the conductive traces. To capture the output voltage and current from the amplifier, a Keysight DSOX2024A oscilloscope (Keysight Technologies (China), Beijing, China) was employed. All signals were recorded using a data acquisition card, ensuring accurate and high-resolution data collection for the subsequent analysis. The process of evaluating the bend and stretch responses of the micro-structured sensors using an LCR meter followed a systematic procedure. First, the LCR meter was calibrated and a sensor based on one of the substrates (TPU, PI, or PET) was securely mounted onto the stretching and bending fixture. Baseline measurements were obtained at zero strain to establish reference values. Subsequently, strain was gradually applied, and at each step, the LCR meter recorded the electrical properties. In the case of dynamic testing, continuous measurements were taken as strain was applied or released at a constant rate, ensuring that the real-time response was captured. Upon completely releasing the strain, final measurements were taken and compared to the baseline values to identify any permanent changes. The outcomes were summarized to determine the impact of strain on the microstructures’ electrical performance.

## 3. Results and Discussion

### 3.1. Surface Structure of Fabricated Sensors

**Figure 2:**
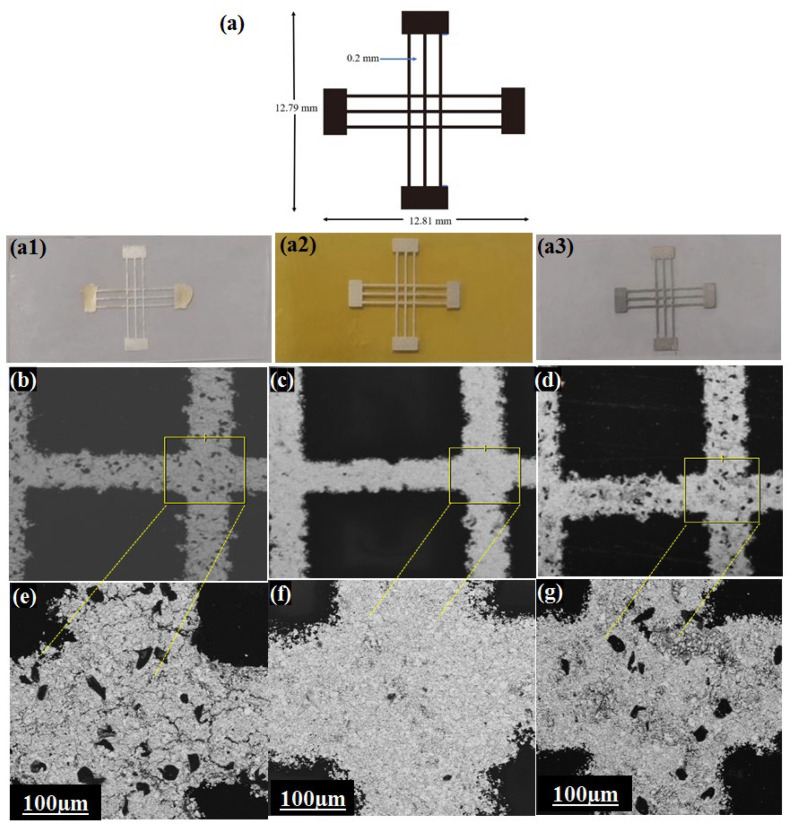
Visualization of the printed silver paste ink lines on different substrates using printing pattern S1. (**a**) Illustration of the printed pattern S1 (real photos on (**a1**) TPU, (**a2**) PI, and (**a3**) n PET); (**b**–**d**) SEM images of the printed silver paste ink on the TPU (**b**), PI (**c**), and PET(**d**) substrates; (**e**–**g**) SEM images of the silver paste ink on TPU (**e**), PI (**f**), and PET (**g**) at a higher magnification.

The surface structure of the fabricated sensors, as observed through SEM images, revealed distinct microstructure characteristics depending on the type of substrate used (TPU, PI, or PET). The thicknesses of the substrates were 300 μm, 25 μm, and 25 μm for TPU, PET, and PI, respectively. An LCR meter was used to measure the electrical performance outcomes and the impact of strain on the screen-printed microstructures. The pattern labeled S1 that was printed on the substrates, along with its dimensions, is illustrated in Figure 2. The dimensions of the pattern were 12.79 mm by 12.81 mm, and featured lines spaced 0.2 mm apart. Figure 2b,e show the characteristics of the flexible sensor fabricated on the TPU substrate with the screen-printed pattern shown in Figure 2a, viewed at different magnifications. This heterogeneous distribution of microstructures was observed in Figure 2e, which also revealed a non-uniform arrangement across the surface. The layered structure observed in Figure 2c,f is from the sensor fabricated on the PI substrate, indicating that the printing ink on the PI substrate had a good smoothness and uniform arrangement, with few printed pores. Figure 2d,g display the characteristics of the flexible sensor produced on the PET substrate, showing the separate printed regions with a non-uniform arrangement, which was smaller than the printed pattern on TPU. These findings suggest that the distribution of printed materials on the substrate was not uniform, which could affect performance.

**Figure 3:**
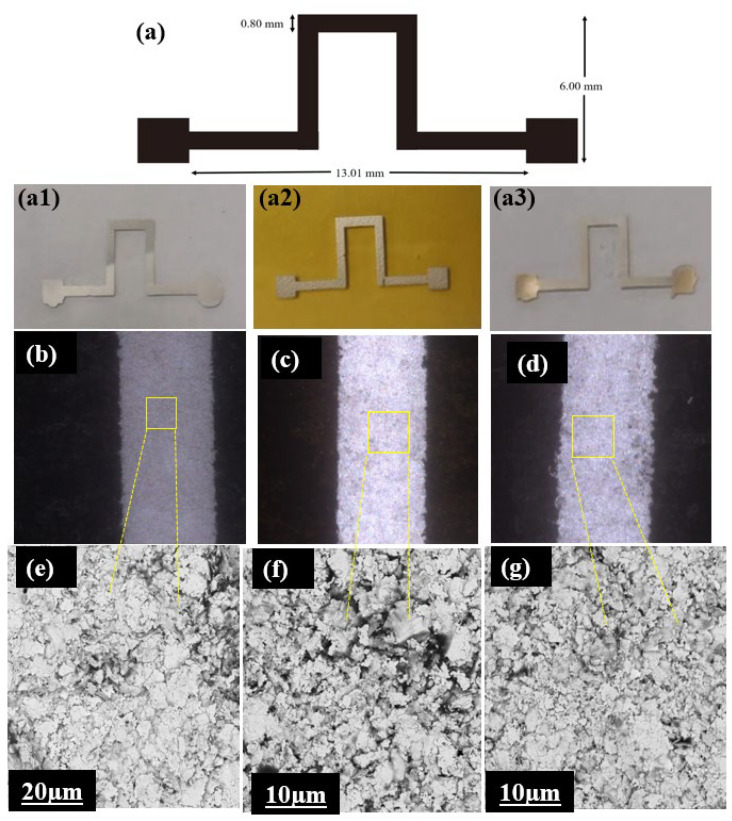
Visualization of the printed silver paste ink structure on different flexible substrates using printing pattern S2. (**a**) Illustration of the printed pattern S2 (real photos on (**a1**) TPU, (**a2**) PI, and (**a3**) PET); (**b**–**d**) microscope images of the printed silver paste ink on the TPU (**b**), PI (**c**), and PET (**d**) substrates; (**e**–**g**) SEM images of the silver paste ink on TPU (**e**), PI (**f**), and PET (**g**) at a higher magnification.

The pattern labeled S2 that was printed on the substrates, along with its dimensions, is illustrated in Figure 3a. Figure 3(a1) shows the fabricated sensors using the TPU substrate; Figure 3(a2) shows the sensors on the PI substrate; and Figure 3(a3) shows the sensors on the PET substrate. Figure 3b–d exhibit distinct regions characterized by differing properties, a coarse arrangement of features, and a complex internal structure. The SEM images in (e), (f), and (g) offer a closer examination of the microstructure in various areas in the TPU, PI, and PET sensors (scale bars: 20 µm (e) and 10 µm (f, g)). These differences in microstructure, visualized through SEM, are essential for understanding how each substrate material interacts with the ink and may affect the overall functionality of the smart sensors.

**Figure 4:**
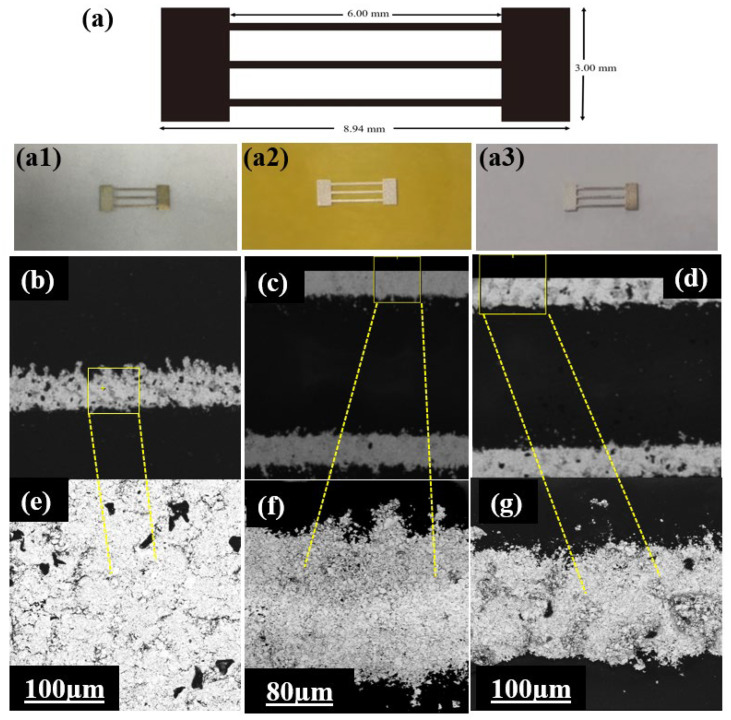
Visualization of the printed silver paste ink lines on different substrates using printing pattern S3. (**a**) Schematic diagram of printed pattern S3 (real photos on (**a1**) TPU, (**a2**) PI, and (**a3**) PET; (**b**–**d**) microscope images of the printed silver paste ink on the TPU (**b**), PI (**c**), and PET (**d**) substrates; (**e**–**g**) SEM images of the silver paste ink on TPU (**e**), PI (**f**), and PET (**g**) at a higher magnification.

The pattern labeled S3 that was printed on the substrates, along with its dimensions, is illustrated in Figure 4a. Figure 4(a1–a3) show photos of the sensors fabricated on the TPU, PI, and PET substrates, respectively. The overall dimensions of the specimen were measured to be 3.00 mm in height and 8.94 mm in length. Figure 4b–d feature microscope images of the printed silver paste ink on the TPU, PI, and PET substrates, respectively. Figure 4e–g show the microstructures printed on the TPU, PI and PET substrates at a higher magnification to allow for detailed observations of their surfaces. Figure 4b,e show a non-uniform arrangement across the surface of the printed area on the TPU substrate, indicating a complex internal structure that could significantly impact the sensor’s behavior. As shown in Figure 4c,f, the silver paste ink applied on the PI substrate exhibited excellent smoothness and a uniform arrangement, with minimal printed pores.

### 3.2. Sensing Performance of Sensors with Printed Microstructures

The stretchability performance of microstructures refers to the ability of these small, designed structures to deform under tensile stress without breaking or losing functionality. This capability is crucial in applications such as flexible electronics, wearable sensors, and biomedical devices. Substrate materials like thermoplastic polyurethane (TPU), polyimide (PI), and polyethylene terephthalate (PET) are preferred due to their flexibility and mechanical strength. Regular designs, such as serpentine patterns or fractal-inspired layouts, are employed to distribute the strain more consistently and prevent restricted failure. Additionally, advanced manufacturing and screen-printing techniques enable the precise construction of these compound structures. The interactions between material selection, structural design, and fabrication technology ultimately determine the overall stretchability, which in turn impacts the reliability and durability of the microstructure devices in practical applications.

After proper calibration of the LCR meter to account for the initial resistance (R0) of the sensors to enable precise calculations of the relative resistance change during stretching, the stretch performances of the sensors were evaluated, specifically when a fixed distance of 100 μm was stretched every 10 s. The sensor was connected to the LCR meter, which was used to measure the change in resistance (ΔR) as the sample was mechanically stretched.

**Figure 5:**
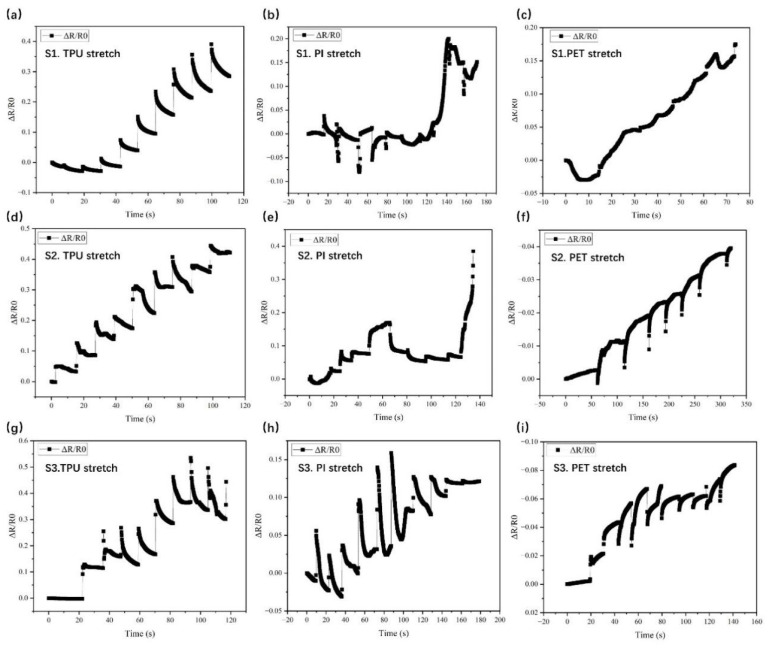
Comparative analysis of stretch performances of sensors on TPU, PI, and PET substrates with different screen-printed patterns. (**a**–**c**) Performance of sensors with pattern S1 on TPU, PI, and PET, respectively; (**d**–**f**) performance of sensors with pattern S2 on TPU, PI, and PET, respectively; (**g**–**i**) performance of sensors with pattern S3 on TPU, PI, and PET, respectively.

The sensor based on TPU consistently displayed a linear increase in resistance across all stretch conditions. As shown in Figure 5a, the TPU-based sensor with structure S1 showed a change in its resistance ratio ΔR/R0 over time (seconds) during the stretching of the TPU substrate. The data exhibited a pattern of incremental increases in ΔR/R0 with time, reaching a maximum value of about 0.40. This periodicity indicates a consistent and regular stretching and relaxing of the material. Figure 5b presents the change in resistance ratio (ΔR/R0) over time for the PI-based sensor during stretching. The data showed variations around the baseline, with minor increases and decreases, indicating less change in resistance compared to the sensor based on TPU. The overall change was smaller, peaking around 0.20 during the stretching process. Figure 5c presents the change in resistance ratio ΔR/R0 over time for the PET-based sensor during stretching. The data showed a relatively smooth increase in ΔR/R0, reaching about 0.18 at stretching time of 75 s.

The stretchability performances of the sensors using the TPU, PI, and PET substrates with fabricated microstructure S2 are shown in Figure 5d–f. Figure 5d shows the change in ΔR/R0 for the TPU-based sensor over time, with a slightly higher maximum value of about 0.45 compared to the TPU-based sensor with structure S1. The incremental steps were more definite, indicating a higher degree of change in ΔR/R0 for the stretching process in each stretch cycle. Figure 5e shows a more obvious increase in ΔR/R0 for the PI-based sensor with structure S2 compared to structure S1 (Figure 5b), with the value reaching up to 0.4. The stretchability performance of the PET-based sensor showed a continuous decrease in ΔR/R0 over a longer duration, reaching about −0.04 (Figure 5f).

The stretchability performances of the sensors based on the TPU, PI, and PET substrates using microstructure S3 are shown in Figure 5g–i. Figure 5g shows the ΔR/R0 for the TPU-based sensor over time, with values reaching up to around 0.55. The incremental increases were more pronounced, suggesting a higher rate of change in ΔR/R0 under the same stretch intensity in each stretch cycle compared to the TPU-based sensor (Figure 5a,d). Figure 5h shows the significant variations in ΔR/R0 during the stretching process, with values ranging up to 0.15. The data indicated a larger recovery of the resistance response to stretching compared to the PI-based sensor (Figure 5b,e). Figure 5i shows a steady decrease in ΔR/R0 for the PET-based sensor over time, with values reaching about −0.10.

Across the above graphs, the TPU stretch data showed a consistent pattern of incremental increases in ΔR/R0. The maximum values increased from approximately 0.40 to 0.45 and finally to 0.55 for microstructures S1, S2, and S3, respectively. The stretch response data for the PI-based sensor showed more inconsistencies and less marked changes in ΔR/R0. Figure 5b,e,h show minor fluctuations around the baseline at the beginning of the stretch process and even more variability, signifying a complex response to stretching. For the PET-based sensor, the data showed a steady and continuous decrease in ΔR/R0 in Figure 5f,i but an increase in Figure 5d.

**Figure 6:**
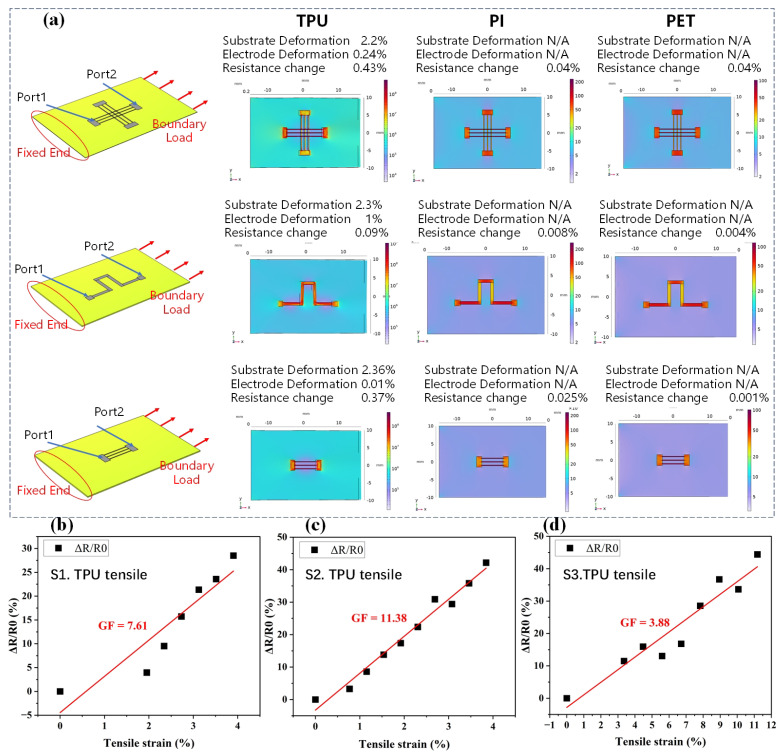
(**a**) Simulation results for TPU, PI, and PET substrates using patterns S1, S2, and S3; (**b**–**d**) resistance responses to tensile strain for the sensors based on the TPU substrate using (**b**) pattern S1, (**c**) pattern S2, and (**d**) pattern S3.

This comparison highlighted the varying sensitivities of the substrates to stretching. The data showed that TPU had more understandable and consistent changes in ΔR/R0 with stretching, while PI and PET exhibited more variability and less predictable changes. The TPU-based sensors displayed the highest change in ΔR/R0 and they had better sensor performances than the sensors using the PI and PET substrates. In order to analyze the mechanism of the sensors with the different substrates, simulations for TPU, PI, and PET were conducted, as shown in Figure 6a. In the simulation, the substrate dimensions were set to 2 cm by 3 cm by 250 μm. One end was fixed and a uniform boundary load of 3 N was applied at the other end. The deformation was calculated based on the displacement in the x-direction. Based on the simulation results, it was concluded that TPU exhibited a significantly better deformation ability than PI and PET, and the electrode deformation of the TPU-based sensor was larger than that of the sensors using PI and PET, indicated that the TPU-based sensor has a better sensor performance under strain.

The resistance responses to tensile strain for the sensors based on the TPU substrate using the different patterns are displayed in Figure 6b–d. When the tensile strain was 4%, the TPU-based sensor with pattern S2 exhibited a response (ΔR/R0) of 42%, and the responses with patterns S1 and S3 were 29% and 30%, respectively. Figure 6b–d provide insights into how the different pattern designs influenced the piezoresistive properties of the TPU substrate. The selection of the optimal patterns for various sensing applications is based on the desired sensitivity, range, and stability of the resistance changes under tensile strain. The different screen-printed patterns (S1, S2, and S3) were designed to investigate how variations in the electrode geometry affect the sensitivity of the strain sensor, i.e., the Gauge Factor. As shown in Figure 6, the Gauge Factors (GFs) were 7.61, 11.38, and 3.88 for the TPU-based sensors with S1, S2, and S3, respectively. It can be seen that the highest GF was from the sensor with screen-printed pattern S2.

**Figure 7:**
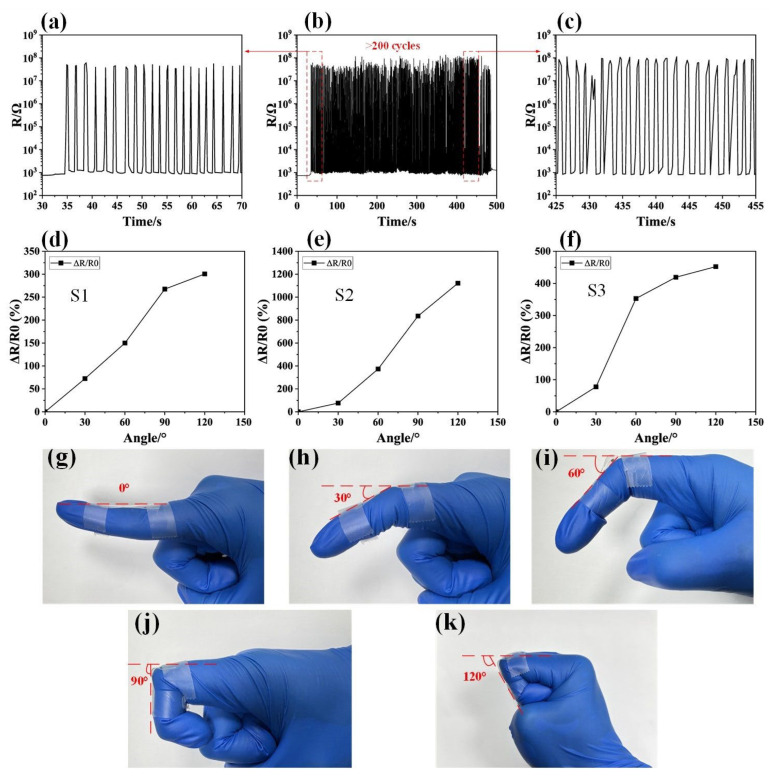
(**a**) The beginning of the durability test, (**b**) the durability test over 200 cycles, and (**c**) the end of the durability test. Finger bending response measurements using the TPU-based sensor with (**d**) pattern S1, (**e**) pattern S2, and (**f**) pattern S3. Photos of finger bending at angles of (**g**) 0° (**h**) 30°, (**i**) 60° (**j**) 90°, and (**k**) 120°.

A durability test with over 200 cycles was conducted; the results are shown in Figure 7a–c. We conducted preliminary mechanical durability tests, including repeated bending over 200 cycles, and found that the sensors showed excellent stability in terms of both electrical performance and structural integrity. Specifically, after the cycles of bending, the sensors demonstrated minimal changes in resistance, which indicates good durability. The TPU-based sensors were attached to fingers through adhesive bonding. The resistance responses of the sensors can be utilized to identify various motion states and bending angles of human joints. As shown in Figure 7d–h, attaching the TPU-based sensor onto the finger area enabled the monitoring of finger bending motion signals for different bending angles of 30°, 60°, 90°, and 120°. When the finger was bent at 30 degrees, the sensor with patterns S1, S2, and S3 exhibited resistance responses of 72%, 76%, and 78%, respectively. The resistance responses of the sensors with patterns S1, S2, and S3 to finger bending at 120 degrees were 301%, 1121%, and 452%, respectively. The TPU-based sensor with patterns S1, S2, and S3 demonstrated similar changes at low tensile strains, while the TPU-based sensor with pattern S2 exhibited a larger response compared to the others at high tensile strains. The TPU-based sensor can effectively collect the motion signals of joints of the human body and could be used in flexible self-powered intelligent wearable devices.

## 4. Conclusions

This research successfully fabricated and assessed screen-printed sensors on thermoplastic polyurethane (TPU), polyimide (PI), and polyethylene terephthalate (PET) substrates. The screen-printing method enabled the low-cost manufacture of durable, conformable sensor structures on these flexible polymer films. The detailed electrical characterization revealed the sensors’ ability to maintain stable performances under various mechanical deformations. The cost-effective fabrication process and use of commercially available substrate materials position these screen-printed sensors for extensive implementation in the field of flexible and wearable healthcare technologies.

Future research should focus on the integration of these screen-printed sensors into comprehensive wearable systems for real-time monitoring of vital signs and physiological parameters. This could involve the development of flexible and stretchable sensor arrays, wireless communication modules, and data processing abilities to create a complete health-monitoring platform. Further optimization of the screen-printing process and exploration of advanced functional materials for the substrate could enhance the sensors’ performance, durability, and range of monitoring capabilities. Overall, this research provides valuable insights into the development of high-performance, mechanically robust, and cost-effective screen-printed sensors on flexible polymer substrates, paving the way for their implementation in next-generation self-health-monitoring systems.

One of the primary challenges in integrating screen-printed sensors into wearable systems is minimizing the power consumption while ensuring continuous and reliable sensor operation. Since these sensors are designed for long-term wear, the power consumption of both the sensors and the signal processing circuits and wireless transmission modules must be improved to the extend battery life. Achieving this balance requires the development of efficient low-power signal-processing circuits and energy-harvesting systems to extend the battery life. Another challenge is the efficient transmission of data from the sensor to external devices, particularly in real-time applications, where delays or data loss could compromise the effectiveness of the monitoring system. Finally, there is the challenge of creating a user-friendly interface that allows patients and healthcare providers to easily interpret the data generated by the wearable system. To address these challenges in future work, we plan to integrate low-power signal-processing circuits, such as energy-efficient amplifiers and analog-to-digital converters, as well as implement energy-harvesting techniques to extend the battery life. Additionally, we aim to incorporate low-power communication protocols, such as Bluetooth Low Energy (BLE) or Zigbee, for efficient data transmission with minimal power consumption. To further enhance the system’s energy efficiency, an adaptive data transmission strategy can be used. This strategy can adjust the sampling rate and transmission frequency based on the monitoring needs and system status. These solutions will be explored in our future work to ensure that the sensor system can be integrated into practical, wearable health-monitoring devices with minimal power consumption and robust data transmission capabilities.

## Data Availability

The original contributions presented in the study are included in the article/Supplementary Materials, further inquiries can be directed to the corresponding author.

## Supplementary Materials

The following supporting information can be downloaded at: https://www.mdpi.com/article/10.3390/mi16030319/s1, Figure S1: Screen printing flow chart: (a) initial state of the machine, (b) polyester mesh stencil for printing, (c) machine-mounted internet board, (d) ink on the polyester mesh stencil, (e) freshly printed ink on the substrate, (f) printed ink after 30 min of drying on the substrate. Figure S2. (a) The contact angles of the TPU, PI, and PET substrates before and after the plasma treatment. (b) Bar chart of the contact angles of the TPU, PI and PET substrates before and after the plasma treatment. Table S1. Comparative analysis of properties of different flexible substrates. References [45,46,47,48,49,50,51,52,53,54,55,56] are cited in the Supplementary Materials.
