# Supplementary material for: Electrical Characterization of Cost-Effective Screen-Printed Sensors Based on Thermoplastic Polyurethane, Polyimide, and Polyethylene Terephthalate
Source: Micromachines (Basel). 2025 Mar 10;16(3):319. doi: 10.3390/mi16030319 (PMC11944745; doi:10.3390/mi16030319)
Supplement: Supplementary file 1 [file micromachines-16-00319-s001.zip › micromachines-3515069-supplementary.pdf]

Supporting Information

Electrical Characterization of Cost-Effective Screen-Printed Sensors Based on Thermoplastic Polyurethane, Polyimide, and Polyethylene Terephthalate

Muhammad Faiz ul Hassan, Yan Wang *, Kai Yang, Yading Wen, Shichao Jin, Yi Zhang and Xiaosheng Zhang

School of Integrated Circuit Science and Engineering, University of Electronic Science and Technology of China, Chengdu 611731, China; faizulhassan858@yahoo.com (M.F.u.H.); 2020050910022@std.uestc.edu.cn (K.Y.); 202221020332@std.uestc.edu.cn (Y.W.); jinsco@163.com (S.J.); yi_zhang@uestc.edu.cn (Y.Z.); zhangxs@uestc.edu.cn (X.Z.)

* Correspondence: wangyanzju@uestc.edu.cn

Here, we provide a more detailed description of the screen-printing process used in our study. As shown in Figure S1, the screen-printing process was carried out using a squeegee to ensure uniform ink deposition without over-saturation. The printing speed was set at appropriately 25 mm/s to achieve precise and consistent patterns using a polyester mesh stencil. A squeegee with a 90 angle was used to push the ink over the mesh stencil. After printing, the conductive silver paste ink was cured at 80°C for 30 minutes to ensure proper curing while maintaining the mechanical integrity of the flexible substrates. The ink viscosity was maintained for optimal flow, and the printed layer thickness was controlled using this parameter. Post-printing, the samples were inspected under a microscope for uniformity and resolution. These parameters were carefully selected based on prior studies and were optimized to ensure high-quality, reproducible results.

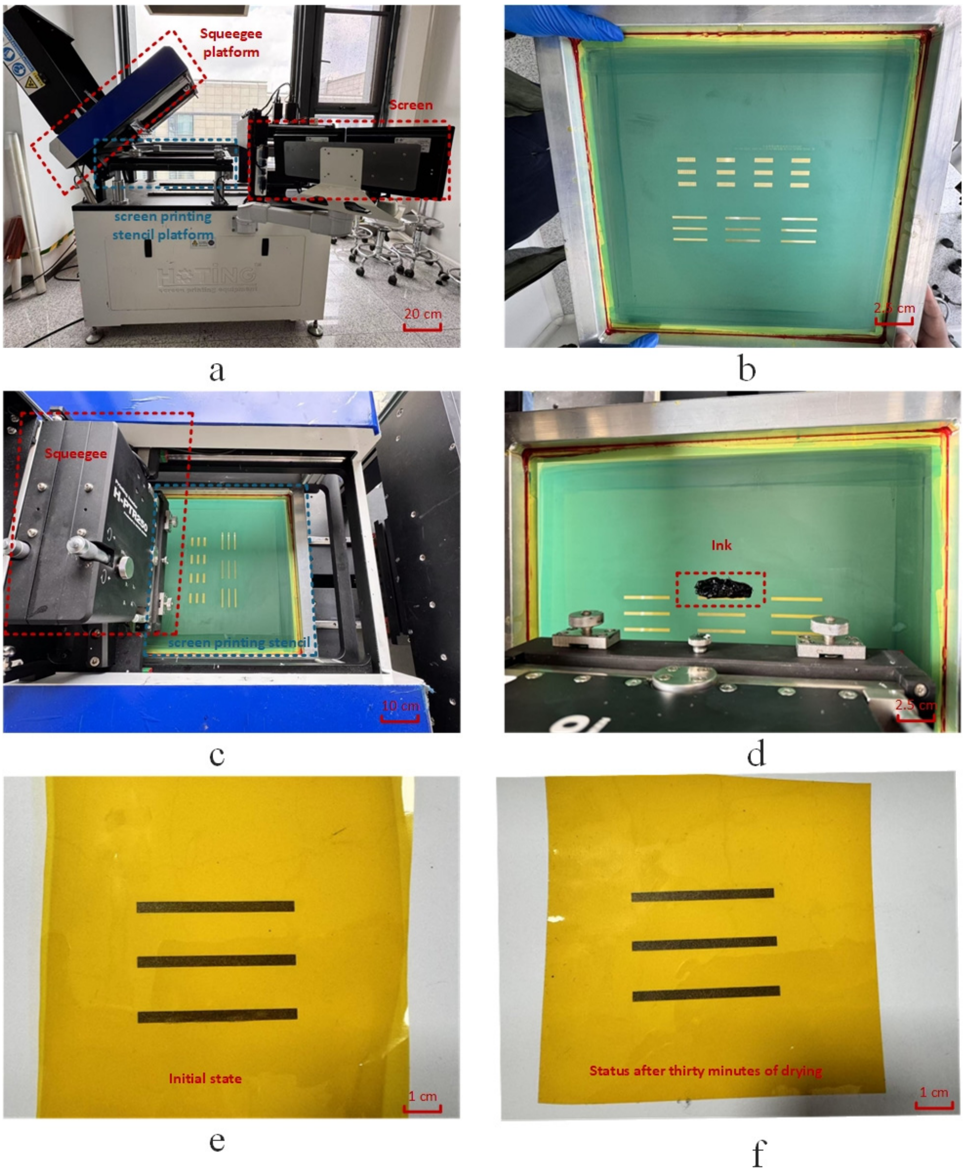

Figure S1: Screen printing flow chart: (a) initial state of the machine, (b) polyester mesh stencil for printing, (c) machine-mounted internet board, (d) ink on the polyester mesh stencil, (e) freshly printed ink on the substrate, (f) printed ink after 30 minutes of drying on the substrate.

To ensure consistency in ink deposition across the PET, PI, and TPU substrates, we meticulously optimized the screen-printing parameters, including squeegee type, speed, and the type of mesh stencil, to maintain uniform ink transfer. The ink's viscoelastic properties, specifically viscosity and surface tension, were carefully controlled to achieve consistent spreading and adhesion on different substrates. Surface pre-treatment techniques, such as plasma treatment and mild heating, were implemented to enhance ink–substrate interactions and minimize variations due to surface energy differences. As shown in Figure S2, the contact angle after the plasma treatment decreased, which indicates that the surface of the substrate became more

hydrophilic after the plasma treatment. The plasma treatment of the materials' surface was conducted before printing process using oxygen at a power of 50 W for 1 minute. The contact angle was measured using a 5 μL droplet of DT water. Additionally, an optical microscopy was employed to monitor the layer condition, ensuring uniform deposition. Multiple deposition runs were conducted under identical conditions to confirm the reproducibility, and the post-printing curing parameters were optimized for each substrate to prevent conductivity and adhesion inconsistencies caused by variations in thermal properties. These measures collectively ensured reliable and repeatable ink deposition across all substrates, thereby enhancing the performance and durability of the printed structures.

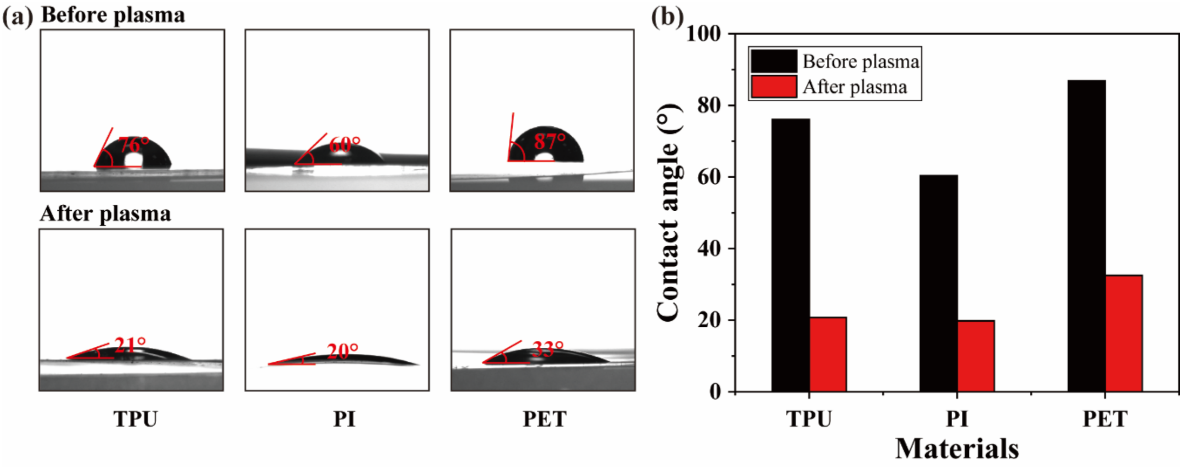

Figure S2. (a) The contact angles of the TPU, PI, and PET substrates before and after the plasma treatment. (b) Bar chart of the contact angles of the TPU, PI and PET substrates before and after the plasma treatment.

The selection of TPU (thermoplastic polyurethane), PI (polyimide), and PET (polyethylene terephthalate) was based on their specific material properties that make them ideal for use in flexible, wearable electronics, particularly for health-monitoring applications. However, it is valuable to consider how they compare with other commonly used flexible substrates such as PDMS (polydimethylsiloxane) and Ecoflex. The comparison of the properties of TPU, PI, and PET with other potential flexible substrates is illustrated in Table S1.

Table S1 shows the comparison of TPU, PI, PET, PDMS, and Ecoflex. We agreed that these data supported the selection of these substrates, especially when compared to the other flexible materials. TPU provides excellent flexibility and a high tensile strength, with extension at break of up to 600%, which allows it to withstand repeated deformation and mechanical stress, ensuring long-term durability [1][2][3]. It is more durable than PDMS and Ecoflex, which are softer and

offer superior stretchability but may suffer from wear and tear over time [4][5]. PI, while more rigid than TPU, offers high mechanical strength and dimensional stability, particularly at high temperatures, which makes it suitable for high-performance applications but with less flexibility [6][7]. PET provides moderate flexibility and mechanical strength, which are more robust than Ecoflex and PDMS, but it is not as flexible as TPU. Overall, TPU, PI, and PET ensure a balance of flexibility and durability, which is critical for maintaining the integrity of wearable health-monitoring devices over long-term use [8][9]. In terms of electrical properties, PI excels due to its excellent electrical insulation, making it ideal for high-performance flexible electronics that require stable conductivity under varying environmental conditions. TPU, while offering moderate electrical insulation, performs well in applications using printed conductive inks, although it does not reach the level of PI in terms of electrical performance [10] [11]. PET provides good electrical insulation, though it is slightly less robust than PI for high-performance applications. In comparison with other substrates like PDMS and Ecoflex, which tend to have lower electrical insulation capabilities, TPU, PI, and PET offer better electrical stability, making them more consistent in providing the proper functionality of wearable sensors over time [11] [12]

Table S1. Comparative analysis of properties of different flexible substrates.

Property	TPU	PI	PET	PDMS	Ecoflex	References
**Flexibility**	Excellent flexibility and stretchability (600%)	Moderate flexibility with high bending resistance	Good flexibility with moderate extension capabilities	Highly flexible	Highly flexible (up to 1000% extension_	[1][2]
**Durability**	Excellent abrasion resistance and long-term wearability	High thermal stability and chemical resistance	Good flexibility with reasonable extension abilities	Low durability	Low durability	[3][4]
**Mechanical Strength**	High tensile strength, suitable for wearable applications	High mechanical strength and dimensional stability	Moderate mechanical strength, good for lightweight	Low tensile strength, soft, and flexible	Low tensile strength, soft, and elastic	[1][2][3][4]

			use			
**Thermal Stability**	Moderate thermal stability, performs well in typical conditions	Excellent thermal stability (up to 400°C)	Moderate thermal stability, good for room temperature use	Low thermal stability, soft at high temperatures	Low thermal stability	[3][4][5][6]
**Electrical Insulation**	Moderate electrical insulation, suitable for flexible circuits	Excellent electrical insulation, ideal for high-performance devices	Good electrical insulation, ideal for standard applications	Not ideal for most wearable applications	Low poor electrical insulation	[7][8]
**Wearability**	Highly comfortable due to its soft, rubber-like texture	Less comfortable, stiffer compared to TPU and PET	Lightweight and comfortable for extended wear	Less comfortable	Comfortable	[9][10]
**Compatibility with Conductive Inks**	Good adhesion with conductive inks, ideal for printing circuits	Good adhesion but more challenging to process due to higher inflexibility	Excellent adhesion, widely used in printed electronics	Moderate adhesion	Moderate adhesion, limited use for electronics	[11][12]
**Chemical Resistance**	Resistant to oils, fats, and many solvents	Excellent chemical resistance	Moderate chemical resistance	Poor, damaged by oil solvents	Poor chemical resistance	[9][10][11][12]

[1] Zhang, Zhennan, et al. "Durable and highly sensitive flexible sensors for wearable electronic devices with PDMS-MXene/TPU composite films." Ceramics International 48.4 (2022): 4977-4985.
[2] Li, Lixia, et al. "A review on polymers and their composites for flexible electronics." Materials advances 4.3 (2023): 726-746.
[3] Li, Bo, et al. "Strain sensing behavior of FDM 3D printed carbon black filled TPU with periodic configurations and flexible substrates." Journal of Manufacturing Processes 74 (2022): 283-295.

[4] Spechler, Joshua A., et al. "A transparent, smooth, thermally robust, conductive polyimide for flexible electronics." Advanced Functional Materials 25.48 (2015): 7428-7434.
[5] Sahatiya, Parikshit, et al. "Graphene-based wearable temperature sensor and infrared photodetector on a flexible polyimide substrate." Flexible and Printed Electronics 1.2 (2016): 025006.
[6] Zhang, Tianyong, et al. "Recent study advances in flexible sensors based on polyimides." Sensors 23.24 (2023): 9743.
[7] Song, Won Geun, et al. "High-performance flexible multilayer MoS2 transistors on solution-based polyimide substrates." Advanced Functional Materials 26.15 (2016): 2426-2434.
[8] Baeg, Kang-Jun, and Jiyoul Lee. "Flexible electronic systems on plastic substrates and textiles for smart wearable technologies." Advanced Materials Technologies 5.7 (2020): 2000071.
[9] Chitrakar, Chandani, et al. "Flexible and stretchable bioelectronics." Materials 15.5 (2022): 1664.
[10] Wang, Yan, et al. "Self-healing conducting composite electrodes derived from chemical recycling of PET plastic wastes for flexible supercapacitors." Polymer Engineering & Science
[11] Ryspayeva, Assel, et al. "A rapid technique for the direct metallization of PDMS substrates for flexible and stretchable electronics applications." Microelectronic Engineering 209 (2019): 35-40.
[12] Lv, Kanghao, et al. "Stretchable carbon nanotube/Ecoflex conductive elastomer films toward multifunctional wearable electronics." Chemical Engineering Journal 500 (2024): 157534.
